# Nicotinic Receptor Alpha7 Expression Identifies a Novel Hematopoietic Progenitor Lineage

**DOI:** 10.1371/journal.pone.0057481

**Published:** 2013-03-01

**Authors:** Lorise C. Gahring, Elena Y. Enioutina, Elizabeth J. Myers, Gerald J. Spangrude, Olga V. Efimova, Todd W. Kelley, Petr Tvrdik, Mario R. Capecchi, Scott W. Rogers

**Affiliations:** 1 Geriatric Research, Education, and Clinical Center, Salt Lake City Veterans Administration Medical Center, Salt Lake City, Utah, United States of America; 2 Division of Geriatrics, Department of Internal Medicine, University of Utah School of Medicine, Salt Lake City, Utah, United States of America; 3 Department of Pathology, University of Utah School of Medicine, Salt Lake City, Utah, United States of America; 4 Division of Hematology, Department of Internal Medicine, University of Utah School of Medicine, Salt Lake City, Utah, United States of America; 5 Associated Regional and University Pathologists, University of Utah, Salt Lake City, Utah, United States of America; 6 Department of Human Genetics and Howard Hughes Medical Institute, University of Utah School of Medicine, Salt Lake City, Utah, United States of America; 7 Department of Neurobiology and Anatomy, University of Utah School of Medicine, Salt Lake City, Utah, United States of America; Centro Cardiologico Monzino IRCCS, Italy

## Abstract

How inflammatory responses are mechanistically modulated by nicotinic acetylcholine receptors (nAChR), especially by receptors composed of alpha7 (α7) subunits, is poorly defined. This includes a precise definition of cells that express α7 and how these impact on innate inflammatory responses. To this aim we used mice generated through homologous recombination that express an Ires-Cre-recombinase bi-cistronic extension of the endogenous α7 gene that when crossed with a reporter mouse expressing Rosa26-LoxP (yellow fluorescent protein (YFP)) marks in the offspring those cells of the α7 cell lineage (α7^lin+^). In the adult, on average 20–25 percent of the total CD45^+^ myeloid and lymphoid cells of the bone marrow (BM), blood, spleen, lymph nodes, and Peyers patches are α7^lin+^, although variability between litter mates in this value is observed. This hematopoietic α7^lin+^ subpopulation is also found in Sca1^+^cKit^+^ BM cells suggesting the α7 lineage is established early during hematopoiesis and the ratio remains stable in the individual thereafter as measured for at least 18 months. Both α7^lin+^ and α7^lin–^ BM cells can reconstitute the immune system of naïve irradiated recipient mice and the α7^lin+^:α7^lin–^ beginning ratio is stable in the recipient after reconstitution. Functionally the α7^lin+^:α7^lin–^ lineages differ in response to LPS challenge. Most notable is the response to LPS as demonstrated by an enhanced production of IL-12/23(p40) by the α7^lin+^ cells. These studies demonstrate that α7^lin+^ identifies a novel subpopulation of bone marrow cells that include hematopoietic progenitor cells that can re-populate an animal’s inflammatory/immune system. These findings suggest that α7 exhibits a pleiotropic role in the hematopoietic system that includes both the direct modulation of pro-inflammatory cell composition and later in the adult the role of modulating pro-inflammatory responses that would impact upon an individual’s lifelong response to inflammation and infection.

## Introduction

Modulation of inflammatory responses by nicotinic acetylcholine receptors (nAChR), ligand gated ion channels permeable to calcium and sodium that are either composed of various combinations of different alpha and beta subunits, is mainly associated to the homomeric alpha7 subtype (α7; [1]). In addition to its role in modulating central neurotransmission, α7 is also expressed by non-neuronal [2] cells including astrocytes, keratinocytes, epithelial cells, adipocytes and those of the immune system including macrophages and lymphocytes [2]–[8]. A function of α7 expression by immune cells is in part to modulate inflammatory responses through affecting the production of inflammatory cytokines as well as chemokines [5], [9]–[14]. For example, upon exposure of skin to ultraviolet radiation α7^KO^ mice exhibit enhanced expression of pro-inflammatory chemokines and cytokines relative to control wild-type mice, and there is a greater influx of inflammatory cells to the exposed tissue of the α7^KO^ mice [12], [14]. Collectively these and similar studies as cited above have shown the null mutation of α7 leads to greater inflammation suggesting that the expression of this receptor is associated with down-regulation of inflammatory responses. However, depending on the tissue and cell type that expresses α7, there can be diverse outcomes. For example, nicotine (a ligand to α7) exacerbates Crohn’s disease while it can ameliorate a certain degree of inflammation in ulcerative colitis [15], also a disease of the intestine. To better understand the mechanisms involved in α7 modulation of inflammation a greater understanding of cells expressing this receptor is required.

Identifying cells that express α7 has been difficult due in part to the relatively low abundance of receptor expression and the poor reliability of commercial reagents used for their detection [16], [17]. Thus, there has been a challenge to clearly identify the cells that express α7 and then determine their relative response when exposed to ligand (e.g., nicotine). Toward resolving this limitation we employed homologous recombination to introduce an *IRES-Cre* bi-cistronic gene cassette after the 3′ end of the mouse α7 gene (*Chrna7*) [18]–[20]. Upon crossing these mice with females harboring the conditional reporter; Rosa26-loxP (yellow fluorescent protein, YFP) [21]–[23], offspring are referred to as α7^Cre:YFP^ [18]. In these mice α7 lineage positive cells (α7^lin+^) are identified by YFP expression using flow cytometric analysis. This has enabled us examine with heretofore unavailable precision and accuracy the participation by α7^lin+^ cells in a pro-inflammatory environment.

In this report we describe the expression of α7^lin+^ cells of the hematopoietic system using the α7^Cre:YFP^ mice. Both lymphocyte and myeloid cells contain populations of α7^lin+^ cells which average 20 - 25% of the total cells. The ratio of α7^lin+^:α7^lin–^ (1∶5) appears to be established early in hematopoiesis as observed by the similar expression ratio in lineage negative cKit^+^Sca1^+^ bone marrow cells. Both α7^lin+^ and α7^lin–^ bone marrow cells functionally reconstitute major immune cell populations when injected into irradiated recipient mice. Further, recipient mice reconstituted with bone marrow cells consisting of 50% α7^lin+^ and 50% α7^lin–^ cells have an equivalent number of α7^lin+^ and α7^lin–^ cells at 8 to 12 weeks post bone marrow transfer (BMT). This result demonstrates that these populations of cells have an equal capacity to re-populate the hematopoietic system. Functionally, in response to LPS challenge, α7^lin+^ cells exhibit significantly enhanced IL-12/23(p40) expression over the α7^lin–^ counterparts. Other cytokines produced by α7^lin+^, as determined by intracellular staining and flow cytometry (FC), include TNFα, IL-6 and IL-10. Collectively these results suggest that the α7^lin+^ identifies a novel hematopoietic cell subtype whose abundance contributes to establishing the inflammatory status of the animal throughout life.

## Materials and Methods

### Reagents

EDTA (Sigma Aldrich, St. Louis, MO), bovine serum albumin, Fraction V (BSA, Roche, Indianapolis, IN), sodium azide (SA, Thermo Fisher Scientific Inc., Waltham, MA), brefeldin A (eBioscience, San Diego, CA), IC Fixation & Permeabilization kit (eBioscience). Antibodies: Ly6G (clone IA8, BD Bioscience Pharmingen, San Jose, CA), CD45R/B220 (clone RA3-6B2, BD Bioscience Pharmingen), CD4 (clone GHK1.5, BD Bioscience Pharmingen), CD8α (clone 53–6.7, BD Bioscience Pharmingen), Ly6C (clone AL-21, BD Bioscience Pharmingen), CD11b (clone M1/70, BD Bioscience Pharmingen), Gr1(RB6-8C5, BD Bioscience Pharmingen), Fc block (BD Bioscience Pharmingen), F480 (clone BM8, eBioscience, San Diego, CA). TNFα (clone MP6-XT22, eBioscience), IL-12/23(p40)(clone C17.8, eBioscience), IL-6 clone MP5-2-F3, eBioscience) and IL-10 (clone JES5-16E3, eBioscience).

### Animals

Mice were kept in accordance with the Guide for the Care and Use of Laboratory Animals and the University of Utah IACUC regulations and guidelines. Studies have been reviewed and approved by the University of Utah IACUC review committee (Approved Protocols #09-07003 and #12-06001). All mice were housed in a pathogen free environment with water and standard mouse chow provided ad libitum. Each experiment used groups of 3–5 mice that were age, gender and strain matched. C57BL/6 mice (stock # 000664) were purchased from Jackson Laboratories (Bar Harbor, ME) as were the CD45.1 mice (B6.SJL-Ptprca Pepcb/BoyJ, stock #002014). The generation and characterization of the α7^Cre^ mice has been described previously [18]. The Rosa26-loxP(enhanced yellow fluorescent protein (YFP)) reporter mouse line were originally from the Jackson laboratory (Stock Number 006148; see [22], [23]).

### Isolation of Cells from Blood, Spleen and Bone Marrow

Lymphoid organs were removed from sacrificed mice and manually dissociated. Bone marrow cells from the femurs and tibias were crushed in a sterile mortar and pestle, red blood cells lysed (RBC lysis buffer, eBioscience), and the cells were passed through 70 µm nylon mesh filters. Viability was routinely assessed by Trypan Blue exclusion and during Flow Cytometry analysis by exclusion of 7-AAD positive cells. Spleen and lymph node cells were isolated following gentle extrusion from the connective tissue and similarly tested for viability with Trypan Blue and 7-AAD.

### Bone Marrow Reconstitution

Reconstitution of lethally irradiated recipient mice (B6.SJL, CD45.1) with donor bone marrow cells (BM cells) from α7^cre:YFP^ mice (CD45.2) was performed as reported previously [22], [24]. Briefly, cells were isolated from the femurs and tibias of donor mice, red blood cells were lysed and the remaining cells passed through a sterile 70 µm nylon cell strainer and washed several times. BM cells were resuspended in saline containing 2% FBS at a concentration of 10^7^ cells per ml. These cells were then sorted with a Becton Dickenson cells sorter (BD FACSAria III) using BD FACSDiva™ software for YFP^+^ and YFP^–^ cells. Isolated cells were assessed for purity, which was greater than 95%, washed with saline/FBS and resuspended to a density of 1 to 2×10^6^ cells per 100 µl. During this process the recipient mice (CD45.1) were exposed to a split dose (2×6 Gy) at a 3 hour interval using a Shepherd Mark I 137Cs source (JL Shepherd and Associates, Glendale, CA, http://www.jlshepherd.com) at a dose rate of 0.8 Gy/minute. Bone marrow cells were transplanted by the retro-orbital route under tribromoethanol (Avertin) anesthesia (1∶80 dilution of 1 mg/ml sterile solution, 0.5 ml/mouse, i.p.) at a dose of 1 to 2×10^6^ total cells in 0.1 ml volume. After cell injection, the recipient mice were placed on acidified, antibiotic water. Water is first adjusted to pH 2.6 with concentrated HCl and then autoclaved. To this 1 liter was added neomycin and then sterilized by filtration. Mice were kept on acidic, antibiotic water for 2 weeks after irradiation and then switched to acidic water without antibiotics for the remainder of time. Analysis of reconstitution was done at 2, 4, 6, 8 and 10 weeks post-transplant.

### Enrichment of Bone Marrow Cells

Cells were isolated from the bone marrow of mice. Cell suspensions were pelleted by centrifugation and resuspended in 40 µl of running buffer per 10^7^ cells. 10 µl of Biotin Antibody Cocktail (Mouse Lineage Cell Depletion Kit; Miltenyi Biotec) was added to the cell suspension and incubated for 10 minutes at 4–8°C followed by washing in 2 ml of running buffer and centrifuged. After the centrifugation, 30 µl of running buffer and 20 µl of Anti-Biotin Microbeads were added per 10^7^ cells. Suspensions were briefly vortexed and incubated for 15 minutes at 4–8°C. Cells were washed twice by adding 2 mL running buffer per 10^7^ cells and centrifuged. The cell pellets were resuspended in 4 ml of running buffer and this cell suspension was passed through a 70 µm nylon mesh filters prior to separation. Lineage committed and lineage negative cells were separated using an autoMACS cell sorter (to achieve a purity of greater than 80%.

### Flow Cytometry Analysis

For flow cytometry (FC) analysis 0.5–1×10^6^ cells were counted, resuspended in 200 µl FACS staining buffer (PBS, 2% BSA, 0.05% EDTA, SA 0.1%) and placed into tubes on ice. For all samples, Fc receptors were blocked using 1 µg/sample Fc block for 15 minutes on ice. A cocktail of monoclonal antibodies directed against Ly6G, Ly6C, CD45R/B220, CD4, CD8a, Gr1, CD11b, or F4/80 were then added to the tubes at a concentration of 0.2–1 µg per 10^5^ cells and incubated with the cells for 30 minutes on ice in the dark. Cells were then washed with FACS staining buffer and filtered through 85 micron nylon mesh (Small Parts Company, Miami Lakes, FL). Thirty-five thousand events were collected using an Accuri Cell Cytometry System (Ann Arbor, Michigan). YFP is detectable in FL1 (530 nm +/−15). Data were analyzed with either CFlow software (Ann Arbor, Michigan) or FCS Express (De NOVO software, Los Angeles, CA). Data analysis was conducted by gating on live nucleated cells. YFP expression by myeloid or lymphoid cells in cells gated for viability was determined. For all calculations the mean represents the average value for all individual animals in the groups examined. Error bars expressed reflect the standard error of the mean.

### Intracellular Cytokine Analysis

At ten weeks post BMT, mice that simultaneously received α7^lin+^ (1×10^6^) and/or α7^lin–^ (1×10^6^) cells were i.p. challenged with 25 µg of *E. coli* LPS (Sigma) in 100 µl Saline. Forty-eight hours later, splenocytes were isolated, resuspended in complete medium at a density 2×10^6^ cells/ml. Brefeldin A (3 µg/ml, eBioscience) was added to the cultures and incubated for three hours at 37°C. After incubation, cells were washed in FACS staining buffer, Fc receptors blocked with blocking antibody (eBioscience) and stained with either anti-CD11b or anti-B220 antibodies for 15 minutes at 4°C. Cells were then fixed, permeabilized (eBioscience fixation/permeabilization kit), and additionally stained with anti-TNFα, IL-12/23(p40), IL-6, or IL-10 antibodies. Twenty thousand live nucleated cells were collected using the Accuri C6 cytometer and analyzed with FCS Express (De NOVO software, Los Angeles, CA).

## Results

### The Expression of α7 during Hematopoiesis Reveals Distinct α7^lin+^ and α7^lin–^ Lineages

**Figure 1:**
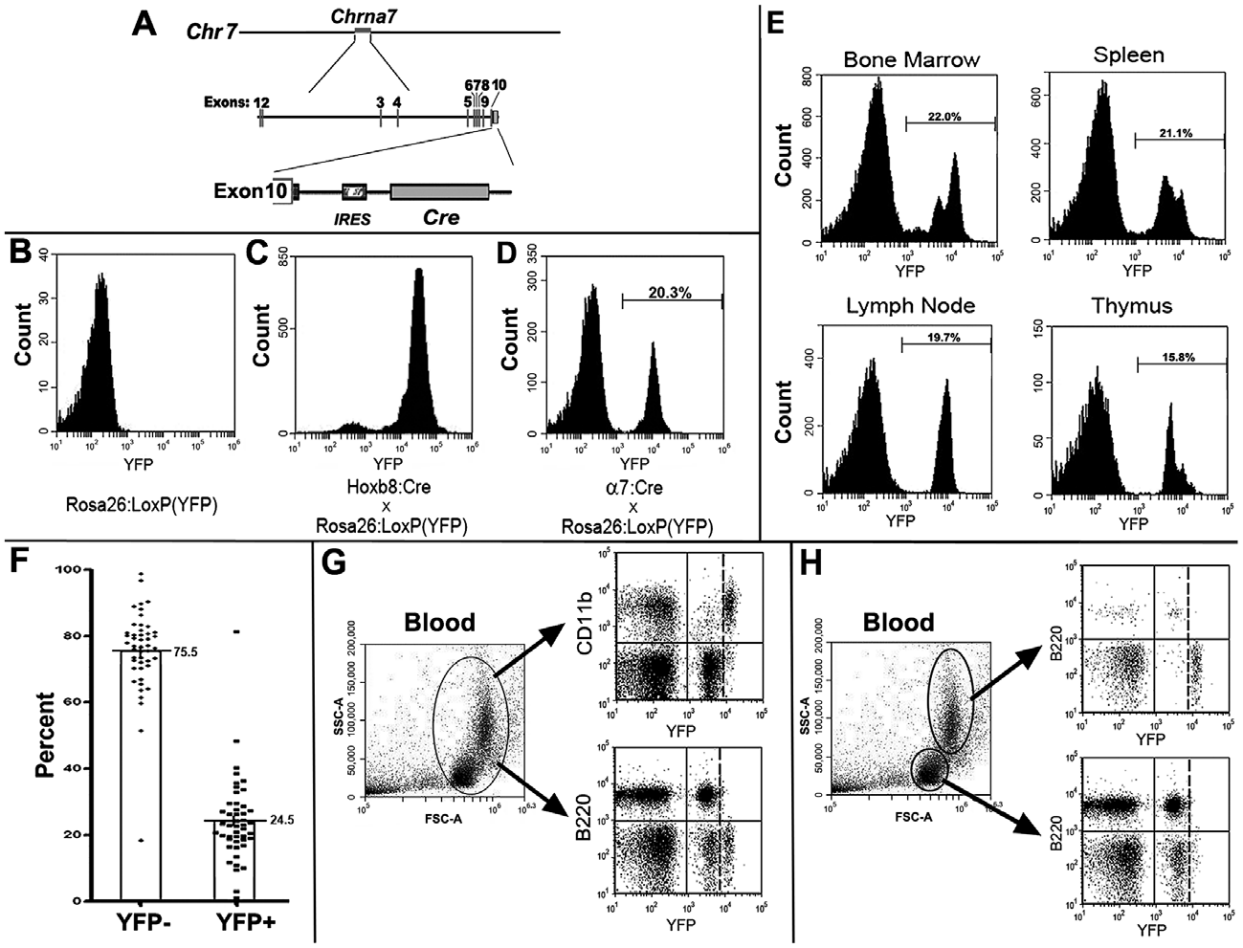
Description of the α7^Cre:YFP^ mouse and distribution of α7^lin+^ cells in lymphoid tissues. A) The α7^Cre:YFP^ mouse [18] was constructed using homologous recombination to introduce into the 3′ end of the α7 gene (*Chrnα7*) a bi-cistronic marker cassette that includes an internal ribosome entry sequence (IRES) and Cre-recombinase (Cre). In these mice, the independent translation of α7 and Cre-recombinase is achieved when *Chrna7* is expressed. B) Bone marrow cells from the Rosa26:LoxP(yellow fluorescent protein (YFP)) parental mice [21], [22] do not express detectable YFP but as in (C) the bone marrow cells from offspring of Hoxb8^Cre^ x Rosa26:LoxP(YFP) crosses exhibit >95% YFP positive cells [22]. (D) In offspring of α7^Cre^ x Rosa26:LoxP(YFP) crosses approximately 20–25% (20% for the example shown) of bone marrow cells from these mice are YFP positive (α7^lin+^). E) Dissociated cells from bone marrow, spleen, mesenteric lymph nodes and thymus of α7^Cre:YFP^ mice were analyzed for YFP expression (α7^lin+^) using flow cytometry. F) A blood sample from each α7^Cre:YFP^ mouse available in our colony (N = 45) was analyzed for α7^lin+^ positive cells. Results are expressed as percent of total cells and the mean value identified. G) Blood samples were further analyzed and a representative plot shows forward scatter (FSC) and side scatter (SSC). Cells identified for YFP expression (X axis) and co-staining for CD11b/Mac-1 or B220 are shown (Y axis). B220^+^/α7^lin+^ are associated with lower intensity YFP while the CD11b^+^ cells are associated with the YFP^hi^. H) Upon setting gates to the indicated ovals (i.e., smaller and less granular cells), YFP^lo^/B220+ cells are distinguished from YFP^hi^/CD11b^+^. These experiments have been performed at least 3 times.

To assess the role of α7 in the induction of innate immune responses a precise determination of the types of cells expressing these receptors is needed. To do this we examined offspring of crosses between the α7^Cre^ male mice ( Methods and Figure 1A and [18] and female mice harboring the ROSA26-LoxP(YFP) reporter (Methods and [21]–[23]). The offspring of these crosses are referred to as α7^Cre:YFP^ mice. Upon bi-cistronic transcription of α7 and Cre recombinase, recombination and expression of YFP is maintained thereafter defining the α7 lineage marked cells (α7^lin+^). Cells not expressing YFP are readily distinguished as (α7^lin–^) cells using FC (Methods). Results in Figure 1B show that YFP is not detected in bone marrow of the Rosa26:LoxP(YFP) control mouse (nor in any other tissue from Rosa26:LoxP(YFP) mouse, including the spleen, not shown). As a positive control for detection of YFP in hematopoietic cells, we isolated cells from the bone marrow of Hoxb8-Ires-Cre x Rosa26:LoxP(YFP) mice [22] that is widely expressed in hematopoietic progenitors. Consistent with this expression, essentially all of the cells from the bone marrow of the Hoxb8^Cre^ x Rosa26:LoxP(YFP) offspring were lineage marked as identified by YFP^+^ expression (Figure 1C). In contrast the detection of YFP in bone marrow cells from the α7^Cre:YFP^ (α7^lin+^) mice (Figure 1D) is approximately 20% of bone marrow cells with the remainder being α7^lin–^. Analysis of cells from several other lymphoid organs (spleen, peripheral lymph node, thymus) shows that like the bone marrow only a portion (15–22%) of the cells in this group were also α7^lin+^ (Figure 1E). A survey using blood samples from α7^Cre:YFP^ mice in our colony (Figure 1F, bar graph) shows that α7^lin+^expression ranged from the extremes of 3% to 81%. However, these mice were rare and the majority of α7^Cre:YFP^ animals express 15–30% α7^lin+^ marked cells (mean = 24.5% +/−1.8 s.e.m.; N = 47). There was no difference in percent α7^lin+YFP+^ cells between young (1–4 months) versus more aged (12–15 months) α7^Cre:YFP^ mice (t-test, p = 0.78; not shown) indicating that this ratio is stable with age. Also, there are no gender related differences in the percentage of α7^lin+YFP+^ cells (t-test, p = 0.25; not shown). For all experiments we used mice expressing 15%–30% α7^lin+YFP+^/CD45^+^ cells (unless specifically noted) as measured in sample blood draws. We have tested red blood cells for YFP expression and have found that these reticulocyte derived cells do not express YFP (not shown). Further, α7^lin–^ and α7^lin+^ sorted cells were tested for α7 gene expression by RT-PCR following RNA isolation. We find expression of α7 transcripts only in the α7^lin+^ cells. No α7 transcripts were detected in the α7^lin–^ cells or in bone marrow cells of the α7^KO^ mouse (using primers spanning exons 8–9; not shown and [12], [14]).

Flow cytometry (FC) results (Figure 1E) often show two peaks of α7^lin+^cells (α7^lin+(hi or lo)^). The relative abundance of these α7^lin+(hi/lo)^ cell populations also differ in their relative proportion based upon tissue distribution where α7^lin+hi^ cells are favored in the bone marrow (Figure 1E), but α7^lin+lo^ dominate the spleen and especially lymph node and thymus populations. This difference in YFP intensity was examined in greater detail. The relative distribution of α7^lin+(hi/lo)^ cells in the blood in terms of forward and side-scatter, and their identity in terms of major cell population markers is shown (Figure 1 G,H). Blood cells that are predominantly CD11b^+^ (Mac-1^+^) account for the majority of the α7^lin+hi^ population of cells (Figure 1G) while B220 expression (B-lymphocyte marker) identifies cells in the α7^lin+lo^ population (Figure 1G,H). This result is also observed for spleen cells (not shown). Further, if cells are gated based on forward scatter (FSC, a measure of cell volume/size) or side-scatter (SSC, a measure of cell granularity), the B220 positive cells are prominently detected in the smaller cells having fewer cytoplasmic granules (Figure 1H) and not the larger, more granular cell populations.

### Distribution of α7^lin+^ Cells in Lymphoid Organs

**Figure 2:**
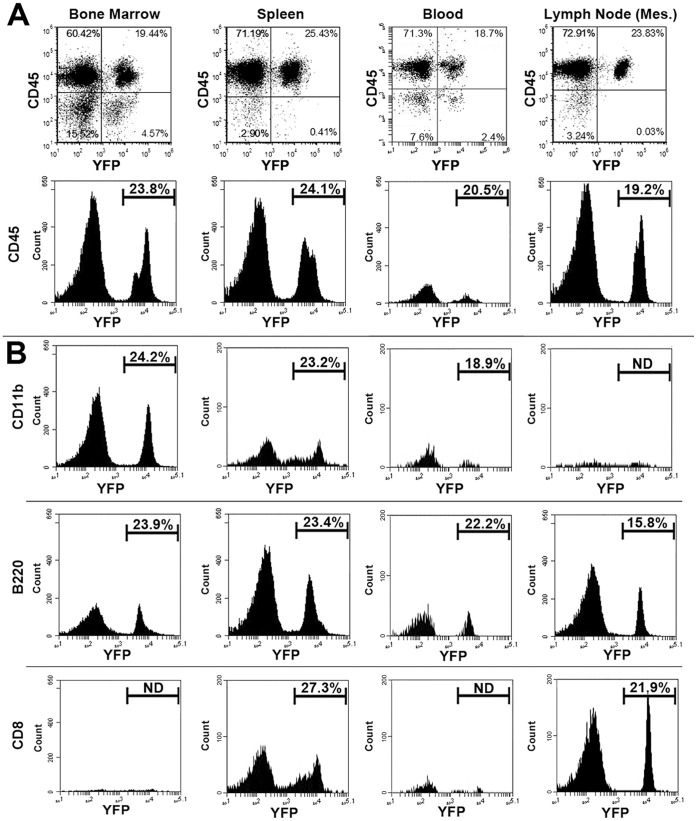
Analysis of cells from lymphoid organs of α7^Cre:YFP^ mouse using markers of CD45^+^ cells. An analysis of α7^lin+^cell subtypes and their distribution in bone marrow, spleen, blood, and lymph node is shown. A) Scatter plots and corresponding histograms of cells from the indicated organ including mesenteric lymph node (Mes). The majority of cells in lymphoid organs are CD45^+^ and the ratio of α7^lin+^:α7^lin–^ is in the 20%–25% range, respectively (see text). B) Gating on CD45^+^ cells, the histograms show the relative distribution of α7^lin+^ cells that are also CD11b^+^, B220^+^ and CD8^+^. CD11b^+^/α7^lin+^ cells were most prevalent in the bone marrow while B220^+^/α7^lin+^ and CD8^+^/α7^lin+^ cells were most prevalent in the spleen and lymph node. A more extensive study of cell markers associated with YFP expression including the inguinal lymph node is shown in Supplemental Figure 1. These experiments have been performed at least 3 times with very similar results.

We next examined α7^lin+^ expression in different cell populations using cell-type specific markers including Gr1 (myeloid, granulocytes), Ly6C (monocytes), Ly6G (neutrophils), CD11b (myeloid), B220 (B lymphocytes), as well as CD4 (T lymphocytes) and CD8 (cytotoxic T lymphocytes) in naïve mice. These results are shown in Figure 2 and in supplemental data (Figure S1). In all lymphoid tissues and cell types examined, the percent of α7^lin+^ cells averaged between 20–25% of the respective cell populations. Cell distributions in different tissues were consistent with expected populations of CD11b^+^ and Gr1^+^ cells in the bone marrow, and lymphocytes (B and/or T) in the spleen and lymph nodes (Figure 2 and Figure S1). Consistent with the results of Figure 1G, H, is that bone marrow had the highest percentage of α7^lin+hi^ cells while spleen and lymph nodes had proportionally more α7^lin+lo^ cells. This result is in accord with the results in Figure 1 showing that B220^+^ B cells were mainly α7^lin+lo^. CD4 and CD8 positive T cells present in the spleen and lymph nodes expressed α7^lin+^ cells and these cells tend to be more α7^lin+hi^. We are further investigating T lymphocyte expression of α7^lin+^ to determine whether the α7^lin+hi^ phenotype is a result of greater α7 expression.

### Analysis of α7^lin+^ in Bone Marrow Precursors of Myeloid and Lymphoid Cells

**Figure 3:**
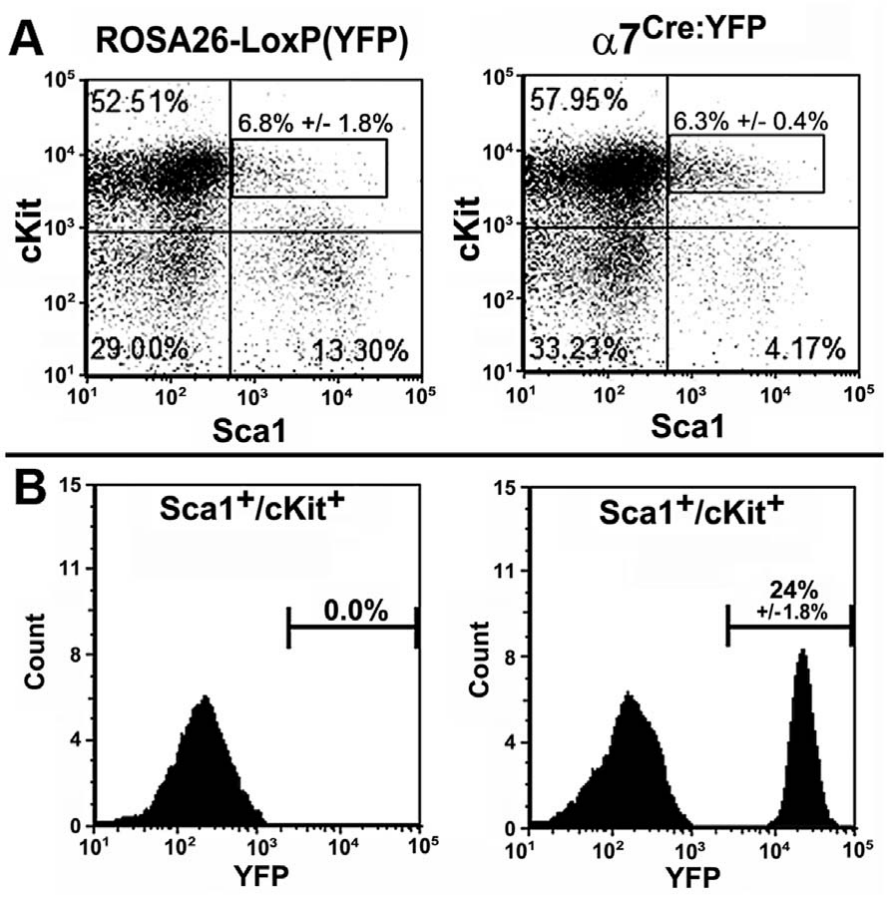
Lineage marker depleted cKit^+^Sca1^+^α7^lin+^ cells are a sub-population of cells identified in the bone marrow of α7^Cre:YFP^ mice. Bone marrow cells from either Rosa26-LoxP(YFP) mice or α7^Cre:YFP^ mice were isolated and depleted of cells committed to a developmental lineage such as myeloid cells (lineage positive cells, see Methods). A) Approximately 6% of the lineage depleted bone marrow cells (boxed) were cKit^+^/Sca1^+^ regardless of their α7 expression background. B) Gating on the boxed cKit^+^/Sca1^+^ cells and measuring the α7^lin+^ or α7^lin–^ cells (FL1/YFP) revealed the absence of α7^lin+^ cells in the Rosa26-LoxP(YFP) control mice, but approximately 24% of the lineage depleted, cKit^+^/Sca1^+^ cells from the α7^Cre:YFP^ mice were α7^lin+^. The percent α7^lin+^ (YFP) positive cells in the bone marrow cells were approximately 18% prior to lineage depletion (not shown). The results shown here represent the averaged data (6.3%) and the standard error of the mean (0.4%) from N = 7 mice.

The observation that the ratio of α7^lin+:^ α7^lin–^ (1∶5) remains stable in mice from birth to approximately 12–18 months old raised the possibility that α7 expression initially arises in a subset of precursor cells of the bone marrow which remain stable. To determine this we examined bone marrow cells based on expression of the cellular markers Sca1 and cKit. Lineage negative cells that are positive for cKit and Sca1 identify a population of hematopoietic stem cells (HSC) that give rise to both mature myeloid and lymphoid cells. Bone marrow cells were first depleted of lineage positive cells by incubation with a cocktail of lineage marker antibodies (see Methods). The lineage negative cells were then stained for Sca1 and cKit [25]. Results shown in Figure 3 show that approximately the same number of Lin^-^Sca1^+^cKit^+^cells (LSK) are present in control mice (Rosa26-LoxP(YFP)) as in the α7^Cre:YFP^ mice (Figure 3A). Further, by gating on the Lin^-^cKit^+^Sca1^+^ cells we determined the percentage of these cells that expressed YFP. As shown in Figure 3B, as expected no cells from the Rosa26-loxP(YFP) control mouse were YFP^+^. On average, 24% (24.3% +/−1.75%, N = 7 mice) of the Lin^-^cKit^+^Sca1^+^ cells from the α7^Cre:YFP^ bone marrow were α7^lin+^ (Figure 3B).

### The α7^lin+^ or α7^lin–^ Bone Marrow Cells Fully Reconstitute the Immune System of Lethally Irradiated Recipient Mice

**Figure 4:**
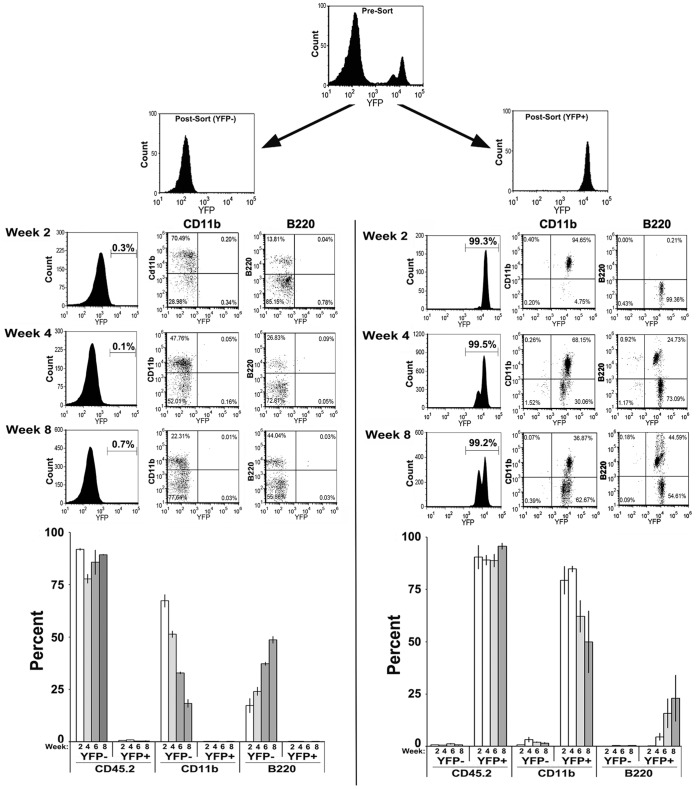
Cell sorting of α7^lin–^ and α7^lin+^ cells from α7^Cre:YFP^ mice followed by injection into lethally irradiated recipient mice results in mice reconstitution by the respective donor population. (A) Bone marrow cells from α7^Cre:YFP^ mice (CD45.2 allele) were dissociated and YFP expression determined by FC. The distribution of α7^lin+^ versus α7^lin–^ cells prior to sorting is shown in the top panel (pre-sort). Following sorting for α7^lin+^ (YFP^+^) or α7^lin–^ (YFP^-^) cells as described in Methods, a sample of cells was analyzed post-sorting as is shown in the left panel α7^lin–^ (YFP^-^) and the right panel α7^lin+^ (YFP^+^). Lethally irradiated recipient mice (CD45.1 allele) were injected with 2×10^6^ of the CD45.2/α7^lin–^ or the CD45.2/α7^lin+^ sorted cells. Mice were monitored via small blood samples for CD45.2 allele to indicate donor specific lineage of cells having cell-type specific markers (e.g., CD11b, B220). Results have been repeated at least 3 times using groups of 4 mice each and these data quantified as shown in the bottom bar graphs (mean +/− s.e.m.). Supplemental data (Supplemental Figure 2) provides a more extensive marker analysis for this experiment including GR1, CD11b, B220, CD8, CD45.1 and CD45.2.

A functional test for α7^lin+^ cells is to reconstitute the immune system of lethally irradiated recipient mice. This approach offers the ability to assess the engraftment of donor cells, as well as the relative efficiency and outcome of reconstitution by α7^lin–^ or α7^lin+^ cells, respectively. To do this, bone marrow cells of α7^Cre:YFP^ mice (which are CD45.2^+^) were collected, dissociated and sorted (see Methods) into α7^lin+^ or α7^lin–^ cells. Recipient mice (CD45.1^+^) were exposed to a total of 12 Gy of irradiation in a split dose (2×6 Gy) on the same day as reconstitution. For each experiment, a total of 6 CD45.1^+^ recipient mice were each injected with 2×10^6^ cells of either α7^lin+^ or α7^lin–^ cells. Blood was monitored at 2 week intervals throughout the experimental period and mice were harvested for cells in immune tissues at 8 to 10 weeks post reconstitution of the recipients. We have evaluated mice for as long as 12 weeks post-reconstitution with equivalent results. In all cases 90% to 99% of the cells analyzed were of donor origin (CD45.2^+^) throughout the experimental period based upon FC analysis of blood sample cells for CD45.1 (recipient) and CD45.2 (donor). The results in Figure 4 show that both CD45.2^+^/α7^lin–^ and CD45.2^+^/α7^lin+^ sorted cells are capable of successful engraftment and establishment of donor macrophages (CD11b) and B-cells (B220; Figure 4) as well as neutrophils and T-cells (Gr1 or Ly6G, CD8, Figure S2). Also evident is that engrafted donor cells that are either α7^lin–^ or α7^lin+^ cells retain their respective phenotypes indicating that CD45.2^+^/α7^lin–^ cells do not initiate expression of α7 during maturation in the bone marrow. As shown in Figure 4, the α7^lin+^ cells that are present at week 2 are predominantly those that express the CD11b marker. With time (as shown for week 4 and 8 post-BMT) there is a shift in the relative proportion of these cells as seen by their overall decrease in percentage that is accompanied by a coordinate increase in the percentage of B220 cells (Figure 4). This suggests that several differences in the engrafted populations do appear between the α7^lin–^ and α7^lin+^ mice over the 8–10 week recovery period. In particular the time course of B-cell engraftment in α7^lin+^ populations is slower and less robust (Figure 4B,C) whereas this group exhibits greater reconstitution of CD11b^+^ (Figure 4) and Gr1^+^ populations (Figure S2) with more prolonged expression in the blood. Supplemental data (Figure S2) shows another experiment with additional cellular analysis for GR1 and CD8 as well as CD11b and B220. Each reconstitution experiment has been repeated at least 3 times with 5 to 6 recipient animals per group.

### The α7^lin+^ or α7^lin–^ Bone Marrow Cells Retain their Identity in Lethally Irradiated Recipient Mice Reconstituted with Equal Numbers of Each Lineage

**Figure 5:**
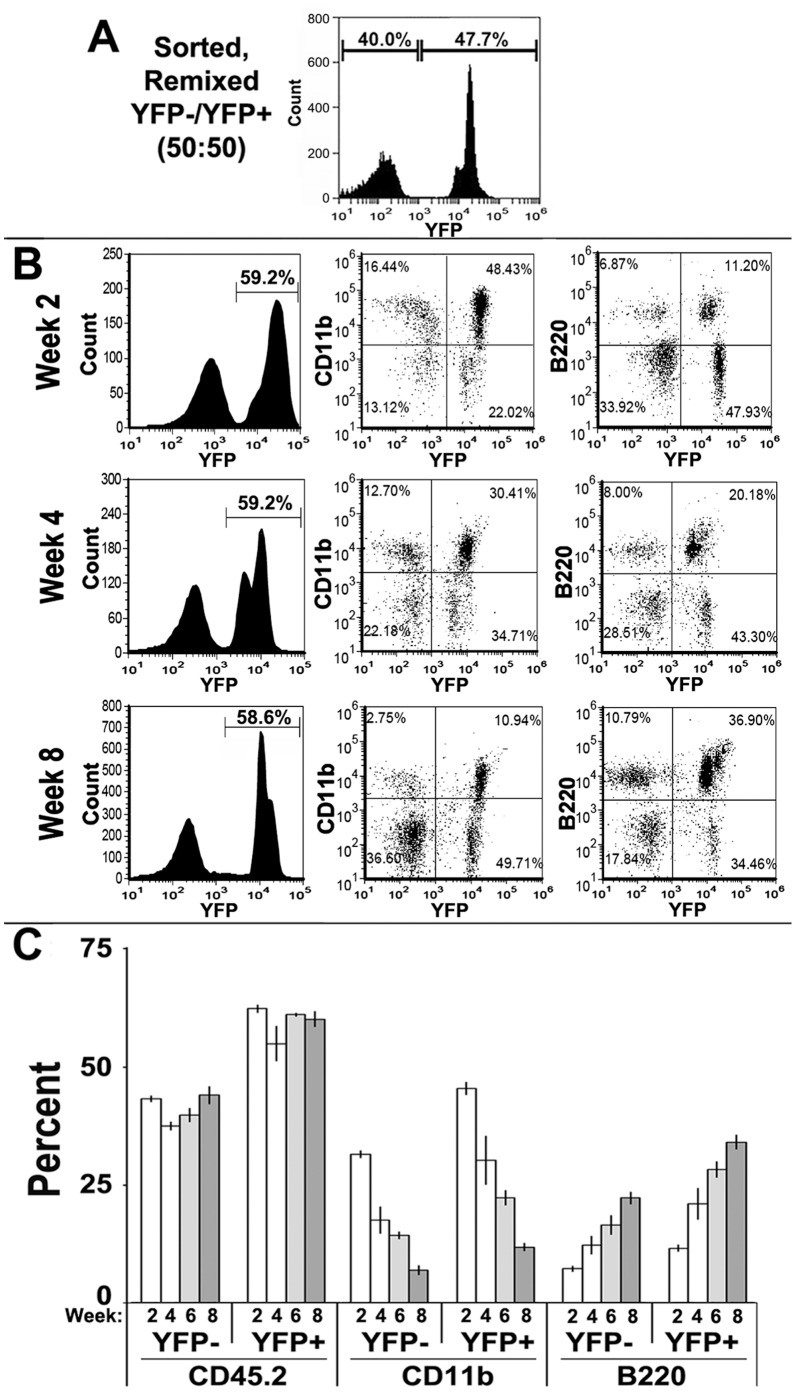
Reconstitution of CD45.1 recipient mice with equal numbers of CD45.2/α7^lin–^ and CD45.2/α7^lin+^ cells results in mice possessing both cell lineages. Upon sorting cells into α7^lin–^ (YFP^-^) or α7^lin+^ (YFP^+^) populations, an equal number of viable cells, were mixed (1×10^6^ α7^lin+^ and 1×10^6^ α7^lin–^). This mixture was analyzed for YFP^−/^YFP^+^ cells by FC which is shown (A). B) Blood samples from these mice were analyzed at two week intervals and assessed for CD45.2/α7^lin–^ and CD45.2/α7^lin+^ cells. The lineage cell markers CD11b and B220 are shown in scatter plots and the percent YFP^+^ of the blood samples is shown in the histograms. A more extensive marker analysis is shown in Figure S2. These experiments have been performed 3 times, 4 animals per group and the quantitation of these findings are graphically shown in (C) (mean +/− s.e.m.).

To further assess the stability of the individual α7^lin+^ and α7^lin–^ phenotypes, we designed the bone marrow reconstitution experiments with the goal of determining whether the α7^lin+^cells were capable of competing with the α7^lin–^ cells upon co-reconstitution. In this case irradiated CD45.1 recipient mice were reconstituted with approximately equal numbers of FACS sorted α7^lin+^ and α7^lin–^ cells (1×10^6^ cells of each phenotype for a total of 2×10^6^ cells). In Figure 5A, re-analysis of the starting viable cell population after sorting and mixing (prior to injection into mice) was 40% α7^lin–^ and 48% α7^lin+^. Analysis of the cellular composition (α7^lin+^ or α7^lin-^) of the mice reconstituted with this mix is shown in Figure 5B. In keeping with more α7^lin+^ cells injected, there a proportional increase in the α7^lin+^ cells at weeks 2, 4, and 8 post-injection (Figure 5B). Two weeks post-reconstitution 59% of the cells in the blood of this recipient were α7^lin+^ (Figure 5 and Figure S2). However, this difference in α7^lin+^ was not evident in all reconstituted mice. In at least 3 repeats of this experiment, the ratio of α7^lin–^ to α7^lin+^ ranged between 40% to 60% α7^lin-^. Data presented in Figure 5C show the combined results of these experiments in bar graphs. Regardless of α7^lin+/−^ phenotype, each population of cells such as CD11b follows a similar pattern of engraftment and reconstitution suggesting that neither of these stem cell populations out-competes the other. This includes the initial robust establishment of CD11b cells and the diminished frequency of this population as well as the increase in B220^+^ cells. Overall, the ability to reconstitute recipient mice is equivalent at this level of examination with variations based more on mouse to mouse differences rather than the differences between α7^lin+^ and α7^lin-^.

**Figure 6:**
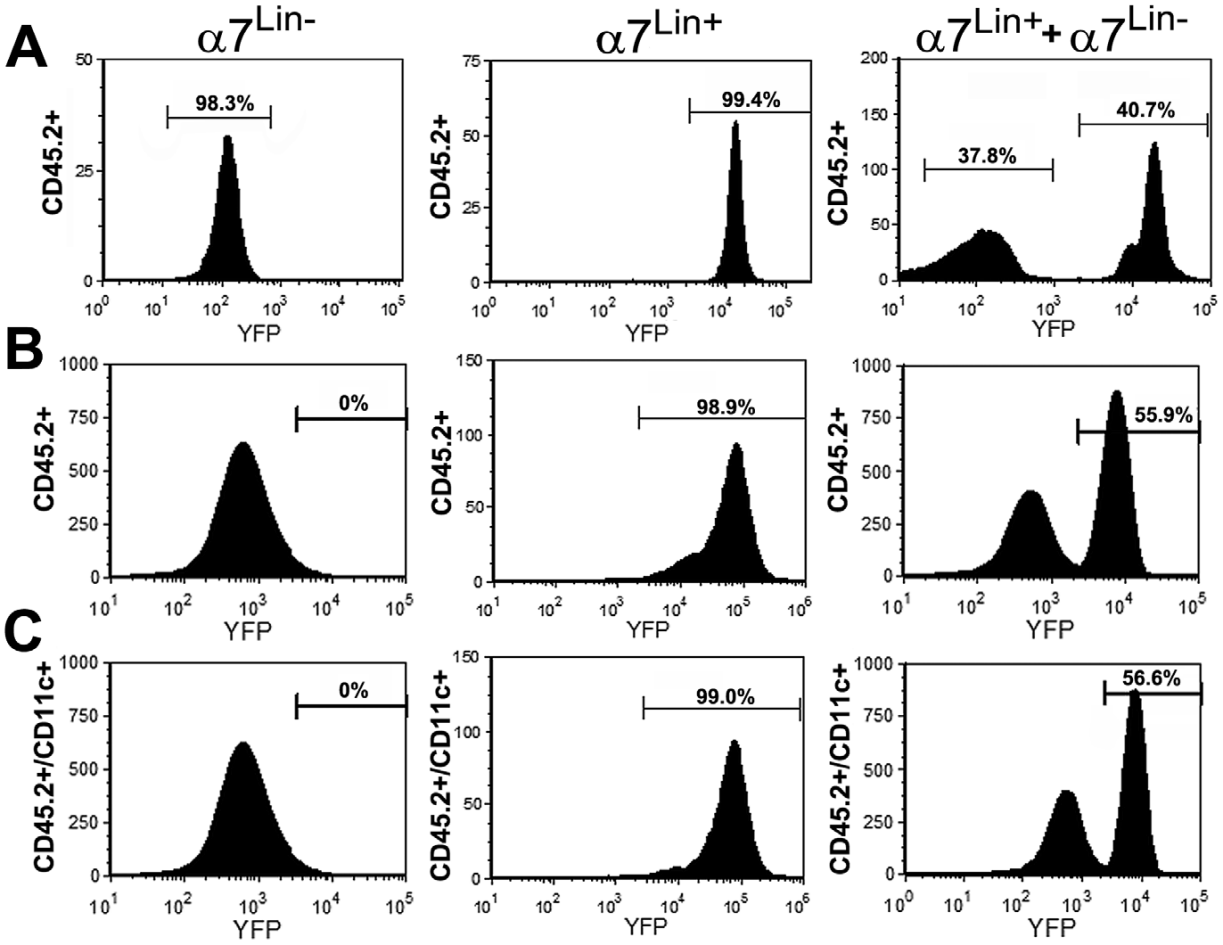
Mice Reconstituted with bone marrow cells that were either α7^lin-^, α7^lin+^ or both, have alveolar macrophages of donor lineage. Bone marrow was isolated from α7^Cre:YFP^ and sorted (see Methods) into α7^lin+^ (YFP^+^) or α7^lin–^ (YFP^-^). In the top panel (A) we show the re-analysis of the sorted cells used to re-populate the recipient mice (CD45.1^+^). These populations were also mixed in even numbers (50∶50) and this mixture injected into recipient mice. Recipient mice were sacrificed at 10 weeks (B) and the α7^lin^ phenotype measured from lung cells collected subsequent to lavage. Lung lavage cells from mice receiving α7^lin–^ cells were YFP^-^, CD45.2^+^ and CD11c^+^ (C) indicating that these cells were AM that differentiated from the donor bone marrow. AM from recipients that were transplanted with α7^lin+^ bone marrow cells were YFP^+^, CD45.2^+^ and CD11c^+^. Lung lavages from recipients of the α7^lin-^:α7^lin+^ CD45.2 bone marrow cells had AM that were all CD45.2 positive and were present in approximately equal numbers. This experiment has been performed at least 3 times with similar results.

To determine whether α7^lin+^ bone marrow cells differentiate into more specialized immune cells in peripheral tissues, we evaluated cells from the lung alveolar space. The majority of resident cells in lung lavage fluid are alveolar macrophages (AM) that are CD11c^+^ and CD11b^-^ [26]. Upon lung lavage of α7^Cre:YFP^ mice we measured via FC α7^lin+^ cells and found that they constitute about 20% of the CD11c^+^ AM (not shown). Results shown in Figure 6 demonstrate the re-population of alveolar lavaged cells following BMT. Recipient mice (irradiated mice possessing the CD45.1 allele) were injected with either α7^lin-^, α7^lin+^, or a 50∶50 mix of α7^lin-^: α7^lin+^ cells following their sorting from the bone marrow of α7^Cre:YFP^ mice. The top panel (Figure 6) shows the re-analysis (FC of CD45.2 and YFP) of the sorted cells prior to injection. Donor bone marrow cells were clearly either α7^lin–^ or α7^lin+^ or α7^lin-^: α7^lin+^ for the 50∶50 mixed cells. Mice were sacrificed at 9 weeks post-reconstitution and lungs were lavaged and analyzed for resident CD45.2 (donor) cells. Results show that upon analysis of isolated cells they were either α7^lin–^ if reconstituted with α7^lin–^ cells, or α7^lin+^ if reconstituted with α7^lin+^ cells. Mice reconstituted with an approximate equivalent percentage of α7^lin–^ and α7^lin+^ cells show slightly more α7^lin+^ cells (56% α7^lin+^ vs. 44% α7^lin-^) in the alveolar lavage fluid although, again, this is not consistent between all experiments. Further analysis of the CD45.2 cells demonstrates that these cells are also CD11c^+^ (lower panel). Not shown is that these cells are also CD206 positive which is also consistent with the phenotype of resident AM. Important in this evaluation (and discussed further below), is the observation that bone marrow cell populations that are initially α7^lin–^ have not been observed to subsequently express α7^Cre^ including during their differentiation into more specialized cells such as AM.

### Inflammatory Response of α7^lin+^ Cells in Post-reconstitution Recipient Mice

**Figure 7:**
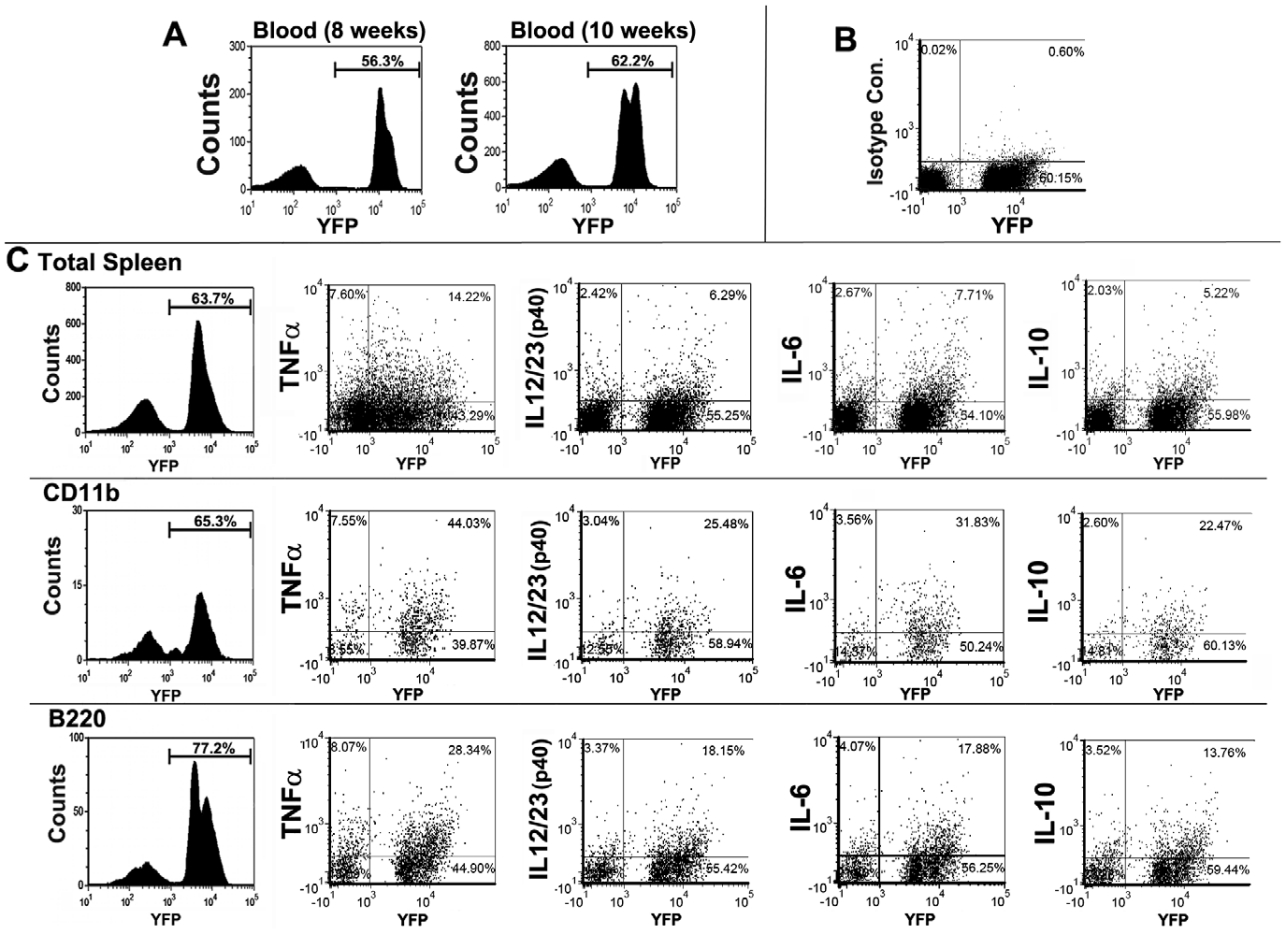
Production of inflammatory and anti-inflammatory cytokines by spleen cells of recipient mice reconstituted with both α7^lin+^ and α7^lin–^ cells. (A) Mice reconstituted with a mixture of 1 x10^6^ α7^lin+^ and 1×10^6^ α7^lin–^ cells were assessed at 8 weeks for blood cells of donor origin (CD45.2) (A, left panel). At 10 weeks post-reconstitution mice were injected with 25 µg of LPS i.p. and the blood of LPS injected mice was analyzed 48 hours later (A, right panel). (B) Isotype control antibodies used for the detection of intracellular cytokines. (C) Flow cytometry of intracellular stained cytokines in spleen cells of α7^lin−/lin+^ reconstituted mice 48 hours post-LPS challenge. Results are expressed as cytokine positive cells in the total spleen cell population (top row), CD11b positive and cytokine positive cells (C, middle row), or B220 positive and cytokine positive cells (C, bottom row). The cytokines examined were murine TNFα, IL-12/23(p40), IL-6, and IL-10. Results have been repeated 3 times.

The lineage mark of cells capable of α7 expression allows us to examine differences in the inflammatory response between α7^lin–^ and α7^lin+^ cells. Following reconstitution of mice with 2 × 10^6^ cells consisting of 1×10^6^ α7^lin+^ and 1×10^6^ α7^lin–^ BM cells we measured cytokine production at 10 weeks post-reconstitution. Figure 7A shows that at 8 weeks post reconstitution there were slightly more α7^lin+^ cells (56%) than α7^lin–^ cells in the blood. Also shown in Figure 7A (right panel) is that at 10 weeks post-reconstitution there were more α7^lin+^ blood cells at 48 hours post-LPS challenge. Spleen cells from these mice were assessed for intracellular cytokines by FC. Spleens from 3 individual animals were harvested 48 hours post-LPS and maintained as distinct samples (i.e., not pooled). Each animal responded in terms of cytokine production by α7^lin+^ and α7^lin–^ cells. Figure 7B shows the isotype controls for the intracellular cytokine antibodies used to assess spleen cells for the cytokines measured (the same isotype control can be used for each cytokine measured, Methods). Results in Figure 7C show cytokine production by spleen cells isolated from the α7^lin-^: α7^lin+^ mice. These analyses show that the cells from the α7^lin+^ cells are functionally active in terms of cytokine production. Further, there is a suggestion that the α7^lin+^ cells are more activated than the α7^lin-^. This result has been observed upon generation of several sets (n = 3 experiments) of α7^lin+^:α7^lin–^ 50∶50 reconstituted mice. Differences between α7^lin–^ and α7^lin+^ cells in the pro-inflammatory cytokine TNFα is accentuated upon gating on CD11b^+^ cells or B220^+^. Other pro-inflammatory cytokines (e.g., IL-6 in CD11b+ cells, IL-12/23(p40) in CD11b^+^ or B220^+^ cells) were also produced by both α7^lin+^ and α7^lin–^ cells. Further, the anti-inflammatory cytokine IL-10 is present in more of the α7^lin+^ cells than in α7^lin–^ cells. These results demonstrate that transfer of bone marrow precursor cells that are α7^lin+^ or α7^lin–^ into irradiated recipients’ results in re-population of the recipient mice with cells capable of functioning as cytokine producing cells.

**Figure 8:**
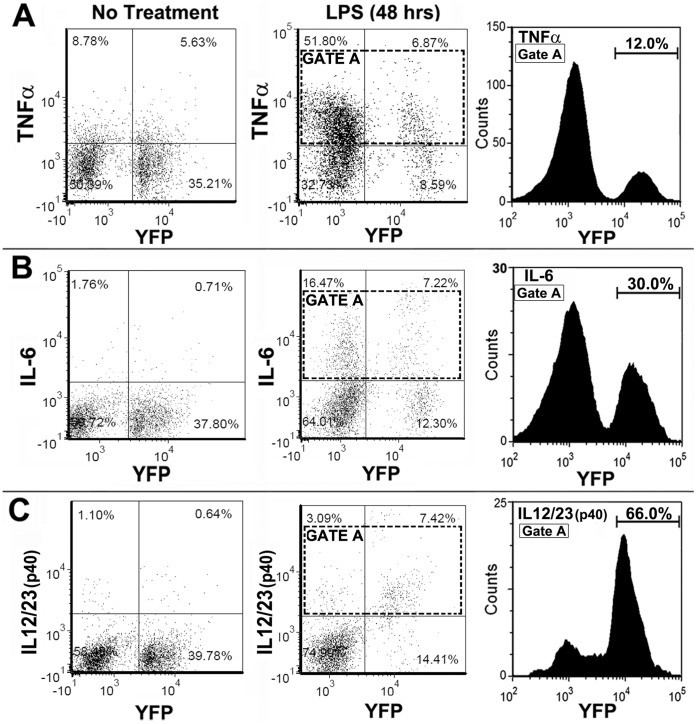
Production of inflammatory and anti-inflammatory cytokines by spleen cells of α7^Cre:YFP^ mice stimulated with LPS. Intracellular cytokine measurements of cells isolated from spleens of α7^Cre:YFP^ mice which were either non-stimulated (no treatment) or LPS injected (25 µg, 48 hr harvest) mice. (A) Intracellular TNFα, (B) Intracellular IL12/23(p40), and (C) Intracellular IL-6. The histograms represent percent of cytokine positive cells (Gate A) following LPS challenge. In all cases, LPS induces cytokine production was increased by LPS indicating a positive response by the animals. Histograms of TNFα^+^ cells (Gate A) show, for example, that the α7^lin–^ cells contribute the majority of the TNFα response of spleen cells to LPS stimulation. Analyses for IL-12/23(p40), and IL-6 are shown in B, and C. IL-12/23(p40) contribution is predominantly from α7^lin+^ cells while the contribution of α7^lin+^ to IL-6 is also substantial (30%). These experiments were repeated 3 times with similar results.

On an equal basis α7^lin+^ cells may produce as much if not more cytokine than α7^lin–^ cells, however there are approximately 5 times as many α7^lin–^ as α7^lin+^ in the α7^Cre:YFP^ mice. Therefore, we questioned whether in the context of the α7^Cre:YFP^ mouse cytokine production by α7^lin+^ cells contributes substantially to the overall inflammatory response. In Figure 8A we show that cells from a naïve α7^Cre:YFP^ mouse have a constitutive level of TNFα expression (8.78% TNFα^+^:α7^lin-^, and 5.63% TNFα^+^:α7^lin+^) which is not unexpected since this cytokine is produced as a precursor protein and stored in the cell. Upon challenge of the mouse with LPS, TNFα is elevated in the α7^lin–^ and α7^lin+^ spleen cells. The α7^lin+^ cells contribute approximately 12% to the total TNFα response (Gate A). Therefore, while these α7^lin+^ cells are functional producers of TNFα, the number of cells expressing this cytokine upon stimulation constitutes a small percentage of the response induced in the animal. Figure 7B shows intracellular staining for IL-6 which suggest that the α7^lin+^ cells contribute IL-6 (30% of the total response in Gate A) to the response but not as much as the α7^lin–^ cells. However, upon analysis of IL-12/23(p40) it is notable that the α7^lin+^ cells contribute 66% of the IL-12/23(p40) response.

These studies demonstrate that; 1) A population of α7^lin+^ bone marrow cells are hematopoietic progenitor cells that can re-populate an animal’s inflammatory/immune system similar to α7^lin–^ cells; 2) The α7^lin+^ and α7^lin–^ bone marrow precursor donor cells retain their phenotype after reconstitution of recipient animals for up to 12 weeks; 3) both α7^lin+^ and α7^lin–^ spleen cells are functional in terms of cytokine production; and 4) α7^lin+^ cells contribute more IL-12/23(p40) to an inflammatory response initiated in the α7^Cre:YFP+^ mouse but also contribute other cytokines such as IL-6 and TNFα.

## Discussion

The innate inflammatory response represents the first line of defense against infectious diseases. However, if an inflammatory response continues unchecked the possibility of undesirable side-effects including chronic inflammation and tissue destruction can be anticipated. Many mechanisms exist for calming the inflammatory response and here we report on the expression of a nicotinic receptor that is reported to have anti-inflammatory effects. There is increasing evidence that agonist (acetylcholine or nicotine) activation of the nicotinic α7 receptor expressed on inflammatory cells modulates the inflammatory response through decreasing the production of cytokines and chemokines [7], [11]–[14], [27]–[30]. This is in part based upon studies that compared the α7^KO^ mice to control WT mice. The α7^KO^ mouse elicits more inflammatory cytokines upon challenge and induces more cellular infiltration into the sight of inflammation. This process appears to, in some cases, be regulated through release of acetylcholine by local parasympathetic vagal efferents to impact upon α7 function and subsequently modulate the function of those cells expressing this receptor such as macrophages [4], [6]. These observations have collectively formed the cholinergic anti-inflammatory hypothesis [4], [31]. However, because similar findings are reported in tissues where parasympathetic innervation is absent, such as the skin, α7 also plays a significant role in modulating the local inflammatory environment presumably due to the local release of acetylcholine by non-neuronal cell types such as keratinocytes [7], [14], [32], [33]. While both vagal and non-vagal mechanisms are consistent with the importance of α7 to impacting upon the magnitude of various pro-inflammatory responses, many questions remain regarding how the expression of α7 influences the system wide inflammatory response and how this can vary between individual animals. Our newly defined mouse models are directly applicable to resolving these issues and they offer the advantage over transgenic models of not interfering with endogenous gene expression or altering normal copy number. Further, as in this study the use of the α7^cre^ mouse crossed with the reporter Rosa26-loxP(YFP) reporter mouse [21] provides a reliable method to identify α7^lin+^ cells through measurement of YFP expression [18], [22], [23]. While the expression of YFP is not necessarily a measure of ongoing α7 expression, α7 transcripts are present in α7^lin+^ bone marrow precursor cells with the anticipated relatively low abundance of 5–10 transcripts per 100,000 β-actin transcripts.

The detection of α7^lin+^ cells in mice reveals that most often between 15% and 25% of the hematopoietic cells are distinguished by this lineage marking. The ratio of α7^lin+^ to α7^lin–^ cells is stable, as reflected by the results of measuring individual animal blood samples over a period of at least one year. In cases where aged animals (>18 months) were available, they too do not significantly deviate from the expectation of ratios defined by younger animals (not shown). Therefore, once established it appears that the representation of the α7^lin+^ ratio does not substantially change throughout the animal’s life. Consistent with this observation is that within the bone marrow, a subset of the Sca1^+^, cKit^+^ cells are also marked as α7^lin+^ progenitor cells. Bone marrow cells containing these progenitor cells (LSK/α7^lin+^) can reconstitute irradiated recipient mice. Reconstitution achieved with bone marrow from mice expressing α7^lin+^ cells also results in approximately the same input ratio of α7^lin+^ to α7^lin–^ in recipients. The possibility suggested in the data that α7^lin+^ cells favor reconstitution of myeloid cells (macrophages and granulocytes) will require further study. We are currently determining the phenotype of the progenitor cells to assess association with α7 lineage. Our results do consistently show that α7^lin–^ cells do not develop or differentiate into α7^lin+^ cells even upon challenge in vivo with LPS. This has functional implications because in bone marrow recipient mice engrafted with equivalent numbers of α7^lin+^ and α7^lin–^ cells, there was a consistent trend for the α7^lin+^ spleen cells (both total and CD11b^+^ cells or B-lymphocytes, respectively) to produce more cytokines upon LPS challenge. Further, α7^lin+^ cells from α7^Cre:YFP^ mice contribute substantially to the splenic IL-12/23(p40) response induced by LPS challenge. This suggests that even though the α7^lin+^ cells constitute on average 20% of the population, the response by these cells to stimulation can contribute approximately 70% of the total production of IL-12/23(p40). Further, the α7^lin+^ cells contribute a significant amount of the IL-6 (>30% of the total IL-6 production) produced following LPS stimulation. The data suggest that the ratio of α7^lin+^ cells in an animal will impact upon the initiation of the physiologic inflammatory response and subsequent responses resulting from IL-12/23(p40) expression. Collectively our findings support the presence of a distinct subpopulation of regenerating cells identified by α7^lin+^ marking that is maintained in both immune (spleen, thymus and lymph nodes) and non-immune organs (lung), and these tend to be more pro-inflammatory than α7^lin–^ counterparts. A biologic significance of α7^lin+^ cells may, in part, be associated with different macrophage phenotypes. Our understanding of macrophage heterogeneity is rapidly expanding and consideration of α7^lin+^ as a marker of macrophage subsets is an intriguing possibility. For example, classically activated macrophages of the described M1 phenotype have a more pro-inflammatory response than those of the M2 phenotype which are associated with wound healing and repair, as well as fibrosis. Whether α7^lin+^ macrophages differentiate into either M1 or M2 macrophage, or if this lineage differentiates into both but predominately into one remains to be determined. Further, if an individual animal has 20% versus 50% α7^lin+^ macrophages that may be of the M1 phenotype, a more pro-inflammatory phenotype in the individual may be present. Similarly, diseases associated with M2 phenotype such as fibrotic diseases may be pre-disposing.

In light of the current understanding of the role α7 in pro-inflammatory responses, our results are somewhat perplexing. We and others have found that the α7^KO^ mouse has an enhanced pro-inflammatory response, demonstrated most often by an increase in TNFα release [13], [14], [34]. We find here that the α7^lin+^ cells are those that are most responsive to stimulation with LPS and contribute substantially to the expression of certain cytokines (IL-12/23(p40)) while having little impact on the total expression of TNFα. Thus the α7^lin+^ cells and the α7^KO^ both exhibit a more pro-inflammatory phenotype, but possibly through the apparent modulation of different cytokines. This result could reflect the complexities of α7 functional pleiotropy that are being revealed for the role this receptor has in directing a variety of developmental processes whose outcome may not be predicted by the functional role this receptor plays in the adult [18]–[20]. First, α7 expression exhibits onset at embryonic day 9 whereupon the pattern expands dramatically thereafter to include multiple tissues and cell types [18], [19]. Many of these sites of expression are transient and vary among specific tissues with widely diverse functions. Thus, to extend this role to this study, it would not be surprising that α7 impacts upon the inflammatory status through different mechanisms including modifying initial conditions of hematopoiesis during development and later in the adult, where α7 has a distinctly different functional role in those cells where expression is ongoing (for discussion and examples see [1], [7], [18]–[20]). The presence of a stable α7^YFP+/−^ ratio from birth, suggests that a likely origin of this lineage marked cell group during early embryonic hematopoiesis is the AGM (aorta-gonads-mesonephros) since α7 expression has not been detected prior to embryonic day E9.0. We are currently investigating this possibility. Second, because the antibody to p40 defines the expression of this subunit in both IL-12 and IL-23, both are being measured. Both of these cytokines have pro-inflammatory functions but IL-23 is distinct in terms of stimulating the induction of Th17 cells whereas IL-12 inhibits this induction. In the α7^KO^ (absence of the α7^lin+^ cells), dysregulated adult cytokine modulatory regulation would lead to the observed net increase in inflammation independently of the IL-12/23(p40) mechanism. Further, α7 is uniquely modulated, both functionally and transcriptionally, by an assortment of external ligands including nicotine and dietary choline (both are α7 agonists and choline is in addition a one-carbon methyl donor; see [18]). Both agents impact on immune and inflammatory responses in the adult [35] and they have a dramatic effect on the early developmental processes where conditional ablation of α7^lin+^ cells leads to multiple birth defects including spina bifida [18]. In addition to the direct functional modulation of the receptor by α7 agonists, *Chrna7* resides in a genomic region well-characterized to be regulated by methylation and parental imprinting [36], [37]. Studies to address many of these issues, such as the mechanism through which the ratio of α7^lin+^ cells is set are in progress. Exposure to ligands of α7 (e.g., nicotine or dietary choline) during development may affect the number and function of the α7^lin+^ cells both *in vivo* and *in vitro*.

Extending the results from previous studies to the present findings suggests that receptor activation and cellular function such as cytokine production [4], [7], [38], and genomic modifications leading to changes in gene transcription are mechanisms that are consistent with the findings from this study. One implication is that environmental-gene interactions both during pregnancy and in the adult would have varied consequences on α7 expression and function that would impact upon an individual’s lifelong response to inflammation and infection.

## Supporting Information

Figure S1 **Identification of α7^lin+^ cells in lymphoid organs from the α7^Cre:YFP^ mouse.** Bone marrow cells, spleen cells, blood, inguinal lymph nodes, and mesenteric lymph nodes were examined for the expression of α7^lin+^ Gr-1^+^ (granulocytes and monocytes), Ly6C^+^ (monocytes), Ly6G^+^ (neutrophils) and CD4^+^. Results show that Gr1^+^ cells are prevalent in the bone marrow, and α7^lin+^Gr1^+^ cells constitute approximately 22% of the total Gr1^+^ cells. Ly6C^+^ cells that are α7^lin+^ compose approximately 25% of the total Ly6C cells in the bone marrow and spleen. The presence of α7^lin+^ cells can be found in all lymphoid organs tested including Peyers patches (not shown). Neutrophils (Ly6G) are prevalent in bone marrow but very few are present elsewhere, and CD4^+^ (T-helper cells) have essentially the same expression pattern and number of α7^lin+^ cells as the α7^lin+^CD8^+^ cells (see Figure 2). These results have been repeated at least 3 times.
(TIF)

Figure S2 **Further identification of donor cell types in the blood of bone marrow recipient mice.** Analysis of blood from recipient mice at various times post reconstitution with donor cells that were either α7^lin+^ (top panels), α7^lin–^ (middle panels), or a 50∶50 mix of the two (α7^lin+^ and α7^lin-^, bottom panels). Figures in the paper show the CD11b^+^ and B220^+^ cells in these mice. Here we show Gr1^+^, CD8^+^ and the CD45.1 recipient post-reconstitution cells that are present at weeks 2, 4 and 8. All cell types are reconstituted with transplantation of either α7^lin+^ or α7^lin–^ cells although α7^lin–^ cells appear best at repopulating CD8^+^ cytotoxic T cells. These experiments have been repeated at least 3 times.
(TIF)
