# Supplementary figures and images for: Nicotinic Receptor Alpha7 Expression Identifies a Novel Hematopoietic Progenitor Lineage
Source: PLoS One. 2013 Mar 1;8(3):e57481. doi: 10.1371/journal.pone.0057481 (PMC3586088; doi:10.1371/journal.pone.0057481)

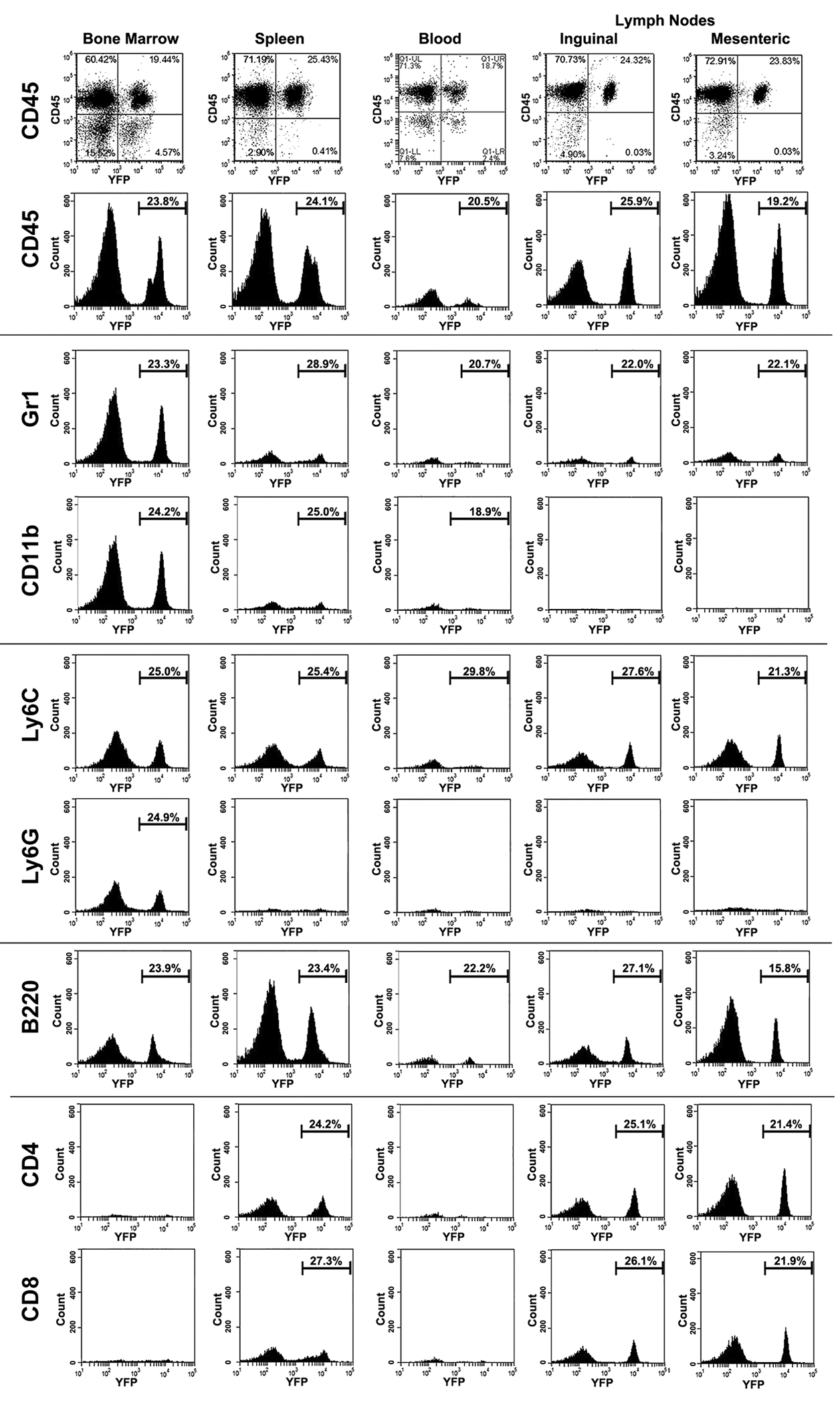

Supplement: Figure S1 — Identification of α7lin+ cells in lymphoid organs from the α7Cre:YFP mouse. Bone marrow cells, spleen cells, blood, inguinal lymph nodes, and mesenteric lymph nodes were examined for the expression of α7lin+ Gr-1+ (granulocytes and monocytes), Ly6C+ (monocytes), Ly6G+ (neutrophils) and CD4+. Results show that Gr1+ cells are prevalent in the bone marrow, and α7lin+Gr1+ cells constitute approximately 22% of the total Gr1+ cells. Ly6C+ cells that are α7lin+ compose approximately 25% of the total Ly6C cells in the bone marrow and spleen. The presence of α7lin+ cells can be found in all lymphoid organs tested including Peyers patches (not shown). Neutrophils (Ly6G) are prevalent in bone marrow but very few are present elsewhere, and CD4+ (T-helper cells) have essentially the same expression pattern and number of α7lin+ cells as the α7lin+CD8+ cells (see Figure 2). These results have been repeated at least 3 times. (TIF) [file pone.0057481.s001.tif]

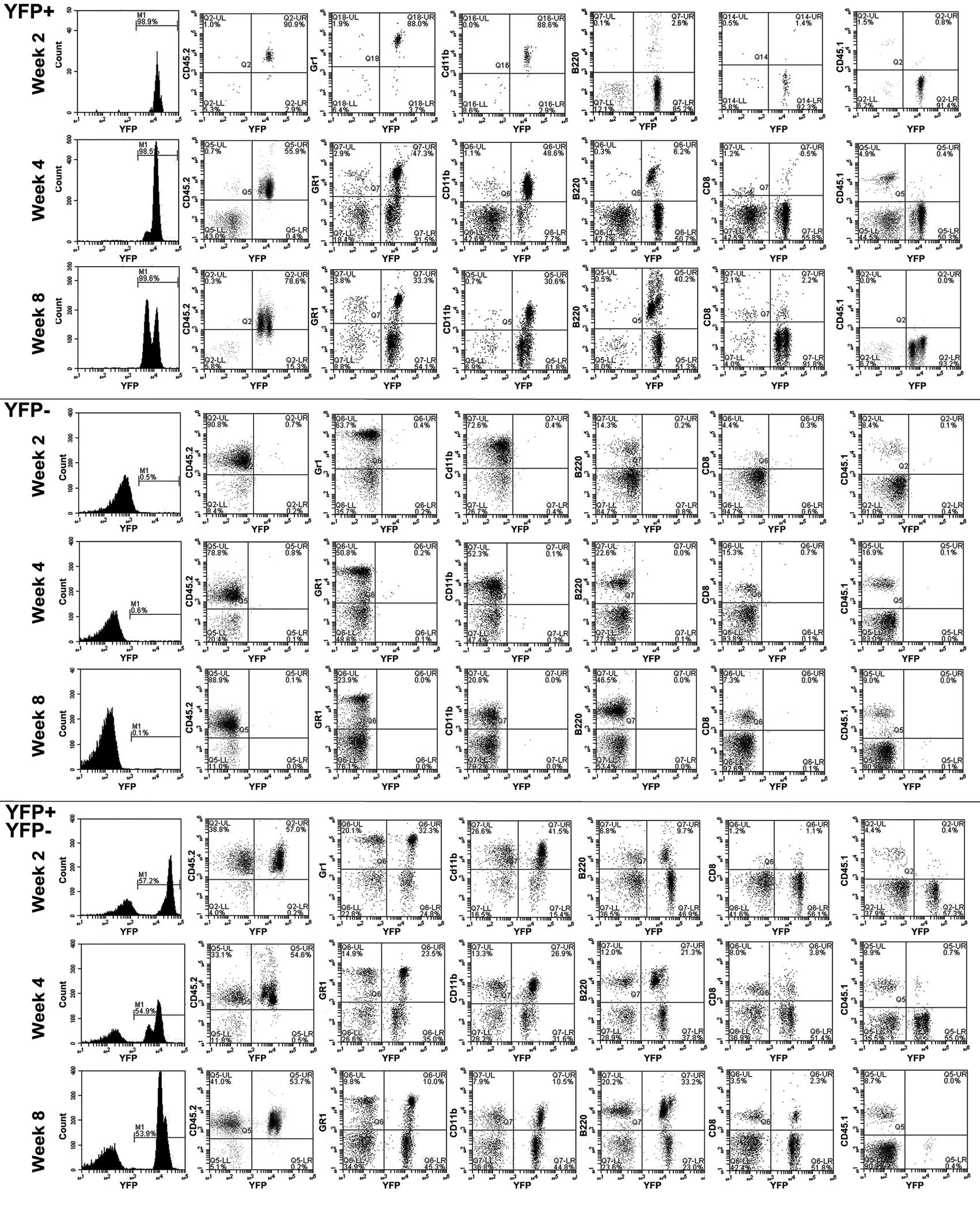

Supplement: Figure S2 — Further identification of donor cell types in the blood of bone marrow recipient mice. Analysis of blood from recipient mice at various times post reconstitution with donor cells that were either α7lin+ (top panels), α7lin– (middle panels), or a 50∶50 mix of the two (α7lin+ and α7lin-, bottom panels). Figures in the paper show the CD11b+ and B220+ cells in these mice. Here we show Gr1+, CD8+ and the CD45.1 recipient post-reconstitution cells that are present at weeks 2, 4 and 8. All cell types are reconstituted with transplantation of either α7lin+ or α7lin– cells although α7lin– cells appear best at repopulating CD8+ cytotoxic T cells. These experiments have been repeated at least 3 times. (TIF) [file pone.0057481.s002.tif]
